# The Prevention of Short Leg in Hip Disease

**Published:** 1898-12-24

**Authors:** 


### THE PREVENTION OF SHORT LEG IN HIP DISEASE.

Mr. Robert Jones, of Liverpool, contributes a paper to the *Lancet* describing the treatment which he adopts with a view to the prevention of short leg in hip disease, and also what he does to rectify it when ankylosis has occurred. There are two points which are of special importance in the first part of this paper. The one is that he insists that the presence of active tuberculous disease in a joint, whether it be the knee or the hip, is no bar to immediate forcible rectification of its position; the other is what he says concerning the advantage of maintaining a position of considerable abduction of the limb in cases where the tendency to shortening is strongly marked. For the latter purpose he has devised a special abduction splint, which is "so designed as to keep the diseased limb fixed in an abducted position, and it effectively prevents any elevation of the anterior superior spine on the sound side; it governs rotation, flexion, and abduction, and in my belief solves the problem of short leg." Let us hope so. This splint appears to be really a double Thomas, with the side for the affected leg set at an angle with the other, and furnished with arrangements for preventing twist of the body. It is obvious, however, to everyone who has to deal much with these cases that the problem to be solved is not merely how to keep the limb abducted, but first and foremost how to get it into that position, and here comes in the importance of what Mr. Jones has to say about forcible rectification. He says that both in his own practice, and in that of Mr. Rushton Parker, "it has always been the custom to rectify with varying degrees of force any tuberculous joint in a wrong position, so far as that phrase might be applied to the deformity known as flexion. . . . In the case of the knee, particularly, I commonly reduce an angle of 30 deg. to 40 deg., and allow the patient to travel home the same morning. . . . In the case of the hip even extensive flexion deformity does not shorten the limb to nearly the extent that adduction with pelvic tilting does. . . . My practice now in all cases of displacement of the head is to at once reduce shortening by force sufficient to effect this purpose. If this be attempted while disease is active and before ankylosis, either fibrous or bony, has had time to take place, scarcely any difficulty is experienced, and pulleys have rarely to be used. . . . Having restored as far as possible the displacement of the hip and the length of the limbs, the patient is placed in the new abduction apparatus," in which he is maintained until the phase of the disease which is productive of deformity has passed. Practically during the whole of the treatment he should live in the open air. From a mechanical point of view, Mr. Jones certainly appears to be right; and in regard to the forcible rectification of position it must be remembered that the experience gained in regard to forcible straightening of the spine in Potts' disease shows that such violence applied to tuberculous conditions is not of necessity so injurious as some of us have been inclined to think.

